# Selections and Abstracts

**Published:** 1898-04

**Authors:** 


### *SELECTIONS AND ABSTRACTS.*

---

### How to Avoid Catching Consumption and How to Avoid Giving it to Others.*

Tuberculosis, or consumption, is an infectious disease which depends for its origin and continuation upon a minute organism known as the tubercle bacillus. This is no longer denied by persons of intelligence.

*If it is an infectious or contagious disease, it is evident that each new case must depend for its origin upon a preceding one.* This self-evident truth is of the greatest importance and must be borne in mind in order to appreciate the force of the following statements:

The cure of this disease is so difficult and uncertain, that up to the present time, we are forced to say that no absolute cure is known.

It is the purpose of the State Medical Society of Washington to alleviate the lot of those afflicted, and to reduce the large mortality from a disease which has increased at a regular ratio in this State from 9.8 per cent. of all deaths in 1892 to about 13 per cent. in 1896. *There are more deaths in the Northwestern States from tuberculosis than from all the reported infectious diseases combined.*

It being granted that consumption is an infectious disease, and that each case results from a preceding one, the lessening of this high death-rate simply resolves itself into preventing contagion, and this is, fortunately, much easier than curing the disease.

It must be understood that tuberculosis is not only a disease of the lungs, but attacks almost every part of the body. It is known under the following names: Consumption, scrofula, marasmus, some forms of meningitis, wasting diseases, white swelling and lupus (a disease of the surface of the body characterized by rapid destruction of the tissues, and so-called from a Latin word meaning a wolf).

---

*Published for gratuitous distribution, by authority of the Washington State Medical Society.

The source of infection in this disease lies solely in the pus, or matter, set free by the sputum when the lung is affected, the discharge from the bowels when the disease is in the abdomen, or the secretion of abscesses or lupus surfaces.

Its entrance to the healthy body is by three avenues: First, the lungs; second, the stomach; third, the skin.

Knowing the source of the disease and its methods of infection, we have the means in our own hands of controlling and practically eradicating it, and that, too, without working hardships upon loved ones, but in reality making life pleasanter and the chances of their recovery many fold greater.

The most fertile source of infection is from the sputum, which, when dried, finds an entrance into the body. *Therefore, all patients must destroy the sputum before it passes from their control.* The following suggestions will be helpful:

1. *At home, expectorate into a cup kept for that purpose.* This cup should be half or three-quarters filled with a solution consisting of one part of carbolic acid to twenty of water. A solution can be made up and kept on hand. The commercial carbolic acid is as good as the refined and is much cheaper. Burn contents and boil cup.

2. *Never expectorate into a pocket handkerchief or cloth which will be allowed to dry.* Keep sputum wet, and best with the above solution. Soak handkerchiefs in the same, and immerse them in boiling water before storing them with the soiled linen.

3. For use upon streets, or when away from home, let patient be provided with thin Japanese napkins. After using, fold up with sputum inside and burn at the first opportunity. A special pocket lined with waterproof material should be provided for these used napkins, and these pockets should be frequently sponged with the above mentioned solution. Napkins can be had at a low price, about one dollar per thousand.

4. Do not spit where domestic animals can have access to this matter. Cattle and fowls are very susceptible and become in turn sources of infection. In fact, do not spit at all where sputum is not destroyed before it can dry

5. Do not spit on streets, and never swallow the sputum.

6. No tuberculous person should kiss any one on the mouth.

7. Tuberculous patients should be smooth shaven. It is impossible to keep a beard clean and from being infected.

8. The tuberculous must always sleep alone.

9. All bed clothing should be changed often (every day when the case is far advanced), and should be at once immersed in boiling water for five minutes.

10. Have separate table utensils and cause them to be scalded as soon as used, and washed separately.

11. Do not permit others to use patient's personal property.

12. A tuberculous mother must not nurse her baby, nor kiss it on the mouth, and in preparing its food must observe special care.

13. Tuberculous persons should not engage in occupations where they are compelled to handle food supplies. If this is unavoidable, use every precaution to prevent infection.

14. Be careful not to infect the sleeping berths when traveling.

There is no need of isolating patients, nor of depriving them of a single home comfort.

Next, a few directions to those who would avoid contracting the disease.

Remember the sources of infection; sputum, bowel discharges and pus from abscesses or tuberculous surfaces.

1. Avoid resorts devoted to the treatment of the tuberculous.

2. Summer and winter, women must wear skirts that clear the walks by not less than four inches, and five or six would be better. Avoid all kinds of fur or other soft trimmings around the lower border of dresses. Americans are expectorating animals, and all the laws in the world and all the good advice that may be offered, will serve only to diminish, but not eradicate this nuisance. Note the filth, especially the sputum on the sidewalks. Skirts dragged through this are taken home, dried, brushed and cleaned, and thus infection is introduced into the household.

3. Do not move into a house where your predecessor was tuberculous, without an efficient disinfection of the premises. To secure such disinfection, have the walls cleaned of old paper and washed with a solution of mercuric chloride (bichloride of mercury) one to one thousand. The woodwork should be painted, after cleaning with this solution, and all floors thoroughly saturated with the same. The solution is a poison.

4. Do not share a consumptive's bed, nor use the personal property, including dishes, belonging to one.

5. Avoid tuberculous food. Fowls and cattle are found to be especially susceptible to tuberculous infections. However, when food is thoroughly cooked infection is destroyed. Milk, especially that for children, must be from cattle free from infection. By heating it to 180 degrees F. for half an hour, it becomes non-infectious.

6. Never put coins or other money into the mouth.

7. Never use a pipe or wind instrument belonging to a consumptive.

8. Probably most important of all; see that the digestive functions are kept in perfect order. Dyspepsia is more often a forerunner of tuberculosis than any other disease. The secretions of a healthy stomach will dispose of a large amount of infected material, but when diseased, the stomach is the principal avenue of infection.

9. Spend as much time in the sunlight and open air as possible. Keep sleeping and living rooms well aired and filled with sunlight. The sunlight acts as a powerful destroyer of the germ.

10. If possible to choose the site of your home, locate it on porous soil. If not, see that the drainings are perfect.

11. Protect all raw or wounded surfaces from any possible tuberculous infection.

12. Do not forget that every new case of consumption comes from a preceding one.

All individuals are not equally apt to acquire tuberculosis. Some are peculiarly predisposed and must be especially careful to follow the above instructions. Such are,

1. Persons born of tuberculous parents, or having several relatives who suffer with tuberculosis in any of its forms.

2. Persons who are enfeebled by privations or excesses. Alcoholic excesses are peculiarly dangerous in this respect.

3. The following may be termed predisposed: All persons suffering or just recovering from measles, whooping-cough, small-pox, influenza.

Those predisposed must not associate with persons afflicted with the disease.

In closing, to quote from a publication of the Pennsylvania Society for the Prevention of Tuberculosis, "Impress indelibly upon your mind that no new case of tuberculosis can arise without an old one. If you can therefore absolutely avoid cases and every source of infection, you are safe whatever predisposing cause you may labor under. With the present prevalence of the disease, however, no one can avoid every source of infection, and it therefore becomes important that predisposing causes as well as sources of infection should be avoided."

---

### THE OBJECTS AND LIMITS OF OPERATION FOR CANCER.

In discussing this subject Cheyne (*British Medical Journal*) says: "The primary object of operation in cancer is, of course, the prolongation of the patient's life and the alleviation of his local trouble; and what I propose to assert in these lectures is that these results are, in most cases, best attained by aiming, whenever it is possible, at the cure of the disease. Until recently, and even now, many surgeons approach operation in these cases impressed with the view that real cure is practically hopeless, and that with few rare exceptions the most that can be expected is prolongation of life for a variable length of time. I therefore hold, and would strongly urge the view, that the first question to be kept before us in investigating a case of cancer is whether there is a possibility of curing the disease or not. Such a point of view makes a great difference in the operation, for it is not then sufficient to remove only the noticeable disease, but it is necessary to take away as far as possible the parts in which the disease may have become disseminated, although still unrecognizable—in other words, possibly infected lymph-areas. Hence it is necessary in all cases where the disease has lasted any time, or extended at all deeply, not only to remove the primary mass freely, but also to take away the whole lymphatic area up to and including the nearest lymphatic glands. Thus the operation performed with the object of curing the disease becomes a much more extensive one, and consequently much more serious than that which simply aims at getting rid of the main trouble for a time and prolonging the patient's life. The first question to be considered, then, with regard to a case of cancer is the anatomical one, namely, whether it is anatomically possible to remove all the local disease and the probably infected lymphatic area so thoroughly as to give a fair chance of non-recurrence. If this is anatomically possible, the next questions are, What are the chances of death as the result of the operation? and What will be the subsequent functional result?

"The primary object of operation in these cases being, therefore, cure, the limit of the radical operation is where there is no reasonable prospect of removing the whole disease, or where, along with a very poor prospect of success, there is a very high mortality from the attempt. In such cases I do not think the operation should be mentioned at all, for even when the patient recovers from it, and has presumably two or three months added to his life, few would, I think, thank one for it, seeing that these two or three months have been spent in convalescing from a serious, and, in the end, useless operation.

"But even in cases where hope of cure or marked prolongation of life by radical operation is out of the question, operation may sometimes be advisable with the object of removing symptoms which are immediately threatening to life—such operations as tracheotomy, colotomy, etc.—or, in the second place, with the idea of taking the primary disease from a part—such as the mouth or throat—where its continued development means intense pain and trouble, and thus of substituting for these troubles an easier death from exhaustion. A *sine qua non* of such operations must, however, be that they are reasonably free from immediate risk; and with regard to the second class, that there is a prospect of attaining the object of the operation, namely, the entire removal of the disease from the part operated upon. I do not think a dangerous operation is allowable for the relief of symptoms, however proper it may be if a cure may be hoped for.

"There are, then, two different objects to be held in view and two different questions as regards operation which we must bear in mind in treating a case of cancer, namely, Can we reasonably hope for a cure? for if we can, a serious or dangerous operation is permissible; or, cure not being possible, Can we decidedly ameliorate the patient's condition by operation, such operation, however, not involving any great danger to life?"

In operations for cancer of the breast the author details and advocates the thorough radical operation, with the removal of the entire breast in all cases, the pectoral fascia, the lymphatic channels, and all the lymph-glands in the nearest groups, including the thorough dissection and clearing out of the axillary glands.

As regards the limits of operation for cure of breast cancer, therefore, he would exclude from operation:

1. Cases of cancer *en cuirasse.*
2. Cases where there is a large mass in the axilla, involving the nerves.
3. Cases where large masses can be felt above the clavicle.
4. All cases where secondary cancer exists elsewhere.

In any cases short of these he believes the patient should be allowed to choose. Even when the operation fails to cure, the prolongation of life is often marked, much more so after the thorough operations than after the ordinary imperfect procedure.

As regards cures—that is, freedom from recurrence for over three years—the author's statistics show that by the radical method of operating which he advocates the number of cures far outnumber, even in the comparatively few cases he has operated upon, the cures recorded by the older operators, which goes to show that this radical form of operation gives not only the best results as regards prolongation of life, but also the greatest proportion of cures. Of his 21 cases there were no deaths; 12, or 57 per cent., cures; 9, or 42.7 per cent., cases recurring externally or internally. This is the result obtained in all operable cases, and not in a selected series of favorable cases.

While the results are steadily improving, the proportion of cases which succumb to cancer is still considerable, and will not, he thinks, be much reduced till patients and doctors understand that there is a good chance of radical cure from early and thorough operation in mammary cancer, and that a suspicious lump in the breast, especially in elderly women, is not a thing to be watched, for over 90 per cent. of the swellings of the breast in elderly women are cancerous.

Contrary to the usual dictum, it is now found that the most favorable of all cases for operation are those of atrophic scirrhus, and the more nearly a cancer approaches the atrophic form the greater is the chance of permanent cure. The author believes that the malignancy of cancer in the individual case has a great deal to do with the favorable result of the operation, more than the early period of the operation.—*Am. Jour. Med. Sciences.*

---

### PURULENT OPHTHALMIA OF THE NEW-BORN.

The following editorial (*Med. Record*) presents this important subject so succinctly and well that it is reproduced entire:

Of the many dangers attending the newly-born infant, ophthalmia neonatorum is the most to be dreaded. It is stated that one-third of all the blind in Europe become so from this cause. Its early recognition and unremitting treatment are of the utmost importance. In every obstetric case the physician should gently separate the lids of the infant, to assure himself definitely of the presence or absence of any eye-discharge. Infection from the maternal passages manifests itself almost invariably by discharge on the third day, both eyes being affected, as a rule. Discharges appearing at a later period usually arise from soiled hands, towels, sponges; and only one eye may be primarily attacked. This conjunctivitis is never due to strong light or cold, as is popularly supposed, but has a definite specific origin.

Following a discussion of the Medical Society of Breslau concerning Crede's method of treating such cases with the aqueous solution of nitrate of silver (one grain to a dram of distilled water), twelve thousand question blanks were sent out to physicians, with results that form an important contribution to the subject. These blanks were distributed throughout Germany, Switzerland, Austria, Belgium and Holland. Reports were returned giving statistics of eye disease in 302,971 new-born infants. Of these 1,938 suffered from ophthalmia neonatorum. In Germany sixty per cent. of eye disease was of this nature; in Austria, eighty per cent.; and in Switzerland, Belgium and Holland combined, ninety per cent. In all these cases the characteristic condition appeared within five days in seventy-six per cent. of the infants under observation. The discharge began after the fifth day in twenty-four per cent. In one-fourth of the entire number of cases one eye was attacked primarily; in three-fourths, both were affected simultaneously. Seventy-one per cent. were completely cured. Nine per cent. discontinued treatment. Twenty per cent., or one-fifth of the 1,938 cases, retained permanent lesions of more or less severity.

This twenty per cent. with permanent ocular defects presented corneal scars, monocular or binocular; and one-half of these permanently damaged infants became totally blind. It was considered they were brought too late for cure; fifty per cent. of the blind babies were not seen until the ninth day of their disease, and twenty-five per cent. until the fourteenth day.

Out of one hundred representative ophthalmologists consulted, seventy-nine were in favor of making Crede's method obligatory in routine obstetric practice. It is not difficult, does no harm, and may avert dreadful catastrophe. The eyes are first carefully washed with tepid water, and the lids thoroughly cleansed by means of absorbent cotton. A few drops of the two-per-cent. solution of nitrate of silver are then instilled into each eye. Materials used to wipe away the discharge must be burnt or otherwise destroyed. Twice daily some simple ointment should be applied to the margin of the lids, to prevent them from sticking together. In severe forms, when there is much swelling and a thick discharge gushing from between the lids, the foregoing nitrate of silver solution may be used every six hours. When the inflammatory reaction subsides, Muskett recommends that weak solutions of alum, sulphate of zinc and perchloride of mercury be substituted. The astringent lotion may consist of from four to ten grains of alum or from one to two grains of sulphate of zinc to the ounce of water. Either of these may be used with freedom and safety. The mercury solution gives excellent results—one-half grain of the perchloride to six ounces of distilled water. Cold-water compresses give great relief after active treatment.

Should ulceration of the cornea ensue, in spite of active and earnest measures, the eserine treatment introduced by De Wecker may prevent perforation in even the worst cases. Four grains of eserine in one ounce of distilled water is the strength usually employed, though sometimes one grain to the ounce is better borne. A few drops are instilled into the eyes six times a day. This treatment is also of value in a form of corneal affection peculiar to infantile purulent ophthalmia, mentioned by Nettleship, in which the cornea becomes rapidly and almost entirely opaque. Whatever the degree of ulceration, marginal ulcers are not so serious as those centrally situated, and sight may be preserved. More than in any other disease, Dr. J. Lewis Smith urges the necessity of employing faithful and attentive nurses, who will carry out punctually the directions given. Two nurses are required, one for day and the other for night duty, since it is essential that the eyes be frequently cleansed and the secretion washed away.—*Med. Standard.*

---

### TREATMENT OF GOUT.

Dr. H. C. Wood has recently, in his characteristic manner, discussed this subject in the *Journal of the American Medical Association.* He says that with regard to drugs there are a great many people who tell us that salicylates do no good. Men do not get good out of salicylates because they do not use them properly. The author does not believe that salicylates cure gout or rheumatism, any more than that bromides cure epilepsy. They simply aid in keeping down the diathesis. If there be any cure, it is exercise. If we use our salicylates on a case properly, and get no response, we have something more than ordinary gout or rheumatism to deal with. There are certain cases which approach typical gout, such as we rarely see in America, in which colchicum does good, much more good than salicylates. He has seen two cases of typical English gout corresponding to Sydenham's description, and only two. We do not have it in this country. These cases colchicum suits better than salicylates do. Sometimes, when the case is on the border line, we will get the best results by a combination of colchicum with salicylates. If we have a strong, robust man, he will stand it. Give him knock-down doses in addition to purging him, and we will bring him through. But that treatment may be worse than the disease, and has to be used with caution.

In using salicylates the profession almost universally choose the worst salt they can find, and that is sodium salicylate. It is, perhaps, not so bad as salicylic acid, but it is much more apt to turn the stomach, and is less effective and more depressing than the other salts of salicylic acid. The two salts which are truly useful are the ammonium salt and the strontium salt. The ammonium salt acts immediately and severely; the strontium salt acts slowly. If we have an acute case we should use salicylate of strontium, or use the two combined. The strontium salt has this advantage, that it does not derange digestion anything like the other preparations, and many a time the author has seen the best effects on the intestinal condition from the use of the strontium salt.

In a large majority of cases we will find that salicylates produce depression, perhaps a little nausea, and general wretchedness, and the patient refuses them. Nine times out of ten we can overcome these effects by combining our salicylate with digitalis and strychnine in the same prescription.

As to baths, we cannot cure a diathesis by such means. But baths are useful—hot baths, steam baths, Turkish baths. Any man who values his own life, who has had a gouty grandfather, ought to take a Turkish bath once a week. We cannot wash out ancestral traces in any other way. The kidney disease and the atheroma would be far less rife if we used the hot bath more than we do. The baths eliminate, give a temporary result, and are very useful when employed with the understanding that they do not cure the disease but relieve the symptoms.—*Therapeutic Gazette.*

---

THE first patent medicine is said to have been called "Tuscarora Rice," sold as a "consumption cure," by Mrs. Masters in 1711. She erected a large factory in New Jersey, and probably inaugurated the patent medicine trade in the United States.—*Oscar Herzberg, in January Lippincott's.*

### HYDROZONE AND GLYCOZONE IN THE TREATMENT OF GONORRHEA.

SIR: My attention has been attracted to an article published in your journal for July 3, by Dr. J. A. Silverman, of Butte, Mont. The writer states that no antiseptic has been discovered that will destroy the gonococcus without doing injury to the mucous membrane. As I presume that he is open to conviction, I submit to you for publication the following report of three cases which I have successfully treated during the last few months with hydrozone and glycozone, which I consider not only harmless but the most powerful healing agents that I have ever used in my practice of thirty-five years.

CASE 1.—A man called on me on June 20, with gonorrhea of four weeks' duration, with profuse discharge, micturition painful, and an acute burning sensation along the entire urethral tract. Pus sacs had formed in the canal, the meatus was inflamed, and the gonococcus was active, as determined by microscopical examination. I prescribed injections of one part of hydrozone and ten parts of sterilized lukewarm water, an ounce for each injection, four times daily. After two days I reduced the proportion to one part of hydrozone and fifteen parts of lukewarm water, and I directed glycozone mixed with an equal amount of glycerin pure to be injected on his going to bed. The diet was not restricted, but no stimulants were permitted. In two days no gonococcus could be detected. The discharge was lessened, the pain and difficulty in micturition had ceased, and in twelve days the patient was well. Continence was imposed for two weeks. Doses of bromide of potassium and bicarbonate of sodium were administered from time to time in order to make the urine alkaline and quiet the patient.

CASE 2.—A married man had contracted blennorrhea from a woman who had the whites. The same treatment was ordered, and with such satisfaction that the woman also was brought for examination and treatment. Result, a cure in each case within three weeks.

CASE 3.—A man, fifty years old, contracted gonorrhea from a woman of the town. As the patient lived in the country, twenty miles out, no treatment was given until ten days after infection. Aggravated symptoms of gonorrhea were present, and there was chordee every night; the patient, to use his own expression, was "plumb wild." The hydrozone injections were ordered, one part to twenty, owing to the great sensitiveness of the urethra and the possibility of orchitis if a stronger injection was used, as there was a slight swelling of the testicles. The glycozone, diluted with equal parts of pure glycerin, was ordered at night. I also gave glycozone internally in medicinal doses, to allay a gastric disturbance due to nervousness. In this case the treatment was continued for twenty-five days. I sent my patient to his cattle ranch happy.—*Warren E. Day, M.D., Prescott, Arizona, in the New York Medical Journal.*

---

### The Tonsil and the Carotid Artery.

At the last meeting of the Laryngological and Rhinological section of the Academy of Medicine, Dr. Robert C. Myles presented an anatomical wet specimen, showing the relations of the carotid and ascending pharyngeal artery to the tonsils. A section was made on each side through the posterior lateral wall of the oro-pharynx, and a string was placed around the artery to bring them into view. It demonstrated a very interesting fact—that the artery in its normal position was situated $1\frac{1}{8}$ to $1\frac{1}{4}$ inches posterior to that part of the palate which was usually selected for the incision in peri-tonsillar abscesses. It was also noted that the artery was situated from one-eighth to one-third of an inch external to a line of incision when made antero-posteriorly. The tonsil was dissected from its basic capsule, which brought into view the peri-tonsillar space, and a rather extensive area in which pus might accumulate anterior to the superior constrictor muscle, and also was so shown the avenue through which pus might burrow under the anterior inferior edge of the superior constrictor muscle. This wet specimen showed that the much feared danger of wounding the carotid artery did not really exist. We often find that there is expressed a fear on the part of those who operate on the tonsils, that the carotid artery may be wounded, but there are by no means many reported cases of this accident. Schmiegelow reports a case occurring in the practice of a surgeon where followed a fatal hemorrhage after the removal of adenoid vegetations. The operation was done without anesthesia, and the ordinary Gottstein angular knife was used. Without any warning a sudden gush of blood issued from the mouth and nose. In spite of prompt tamponing and subcutaneous and intravenous injections of saline solutions the patient died within a few minutes. The internal carotid artery was found to have been opened just in front of its point of entrance into the carotid canal of the pars petrossaossis temporis. The author supposed that swollen glands had pushed the vessel forward, so that the pressure of the knife caused its rupture, for it was not cut.—*Medical Fortnightly.*

---

### SHOULD CITIES GO INTO THE DRUG BUSINESS?

This is the burning question to-day, and the medical press is taking up the question in a manner that leaves no doubt. It is well known that Boards of Health, municipal and State, have sent circulars broadcast to the profession offering antitoxin free to any one who may need it. This is not particularly reprehensible when taken alone. But when these same boards go into the manufacturing business, making antitoxin at a cost to a municipality or State which is greater than it could be obtained from legitimate manufacturers, it is simply a fraud and an imposition on the taxpayers.

This is not the only question, however. A board has no right to enter into commercial enterprises, and this is certainly one of them. It might just as well start a vaccine farm or go into the business of furnishing pure foods and supply the poor with pure milk. The manufacture of antitoxin has been gone into extensively by the New York Board of Health, and, as a natural result, the entire pharmaceutical and a large proportion of the medical profession has expressed its disapproval of this action.

It is unnecessary to go into this question at length. Were it purely from economical grounds alone, antitoxin should be bought from legitimate manufacturers of pharmaceutical goods. From the standpoint of fairness and justice, no municipality should ever try to be a rival of a legitimate manufacturing concern; and from the standpoint of trade honor, it has no right whatever to do such a thing.—*St. Louis Med. and Surg. Journal.*

---

### MORPHINE ANESTHESIA.

Dr. John A. Wyeth, in speaking of anesthetics, says: "The use of morphine as an anesthetic agent has not received the attention it deserves. I removed a larynx on one occasion without the use of a trachea tube, and with complete anesthesia, by producing a profound narcosis by the hypodermic use of morphine. I gave the patient two ounces of whisky half an hour before, and repeated this five minutes before operation; twenty minutes before operation one-quarter of a grain of morphine hypodermically, and an additional one-quarter of a grain was administered five minutes before the operation was begun. During the course of the operation, which lasted one hour and forty minutes, an additional eighth of a grain was given. The patient was entirely free from pain throughout. The antidotes to morphine poisoning (atropine sulphate, gr. $\frac{1}{100}$, strychnine sulphate gr. $\frac{1}{20}$) should be in hypodermic syringes ready for use, and strong coffee for rectal injection."—*Med. Review of Reviews.*

---

### THE QUESTION OF THE TRANSFORMATION OF CALOMEL INTO CORROSIVE SUBLIMATE IN THE ORGANISM.

*Lyon médical* for February 20th says that this question was raised not long ago in the Paris Therapeutical Society. M. Patein stated that such transformation in the presence of sodium chloride, accepted by Mailhe, was a fable; it was not true that calomel was changed into corrosive sublimate on contact with the alkaline chlorides or with the gastric juice. M. Pouchet added that the bromides and the chlorides were powerless to convert calomel into corrosive sublimate; such a change took place only on contact with the alkaline iodides. If it did take place in the presence of chlorides, he said, it could not be avoided by the patient's simply abstaining from salted articles of food; it would be necessary to remove all the chlorides from the organism.—*N. Y. Med. Jour.*

### THE MEDICAL EXCURSION IN JUNE TO DENVER AND SALT LAKE CITY.

The American Medical Association meets at Denver June 7th to 10th. One of the features of the gathering will be an excursion from Denver to Salt Lake City and return via the D. & R. G., Colorado Midland and Rio Grande railways, through the "heart of the Rockies," furnishing a splendid opportunity to view the most magnificent scenery on the American continent. Salt Lake City is an ideal summer resort, and the bathing at Saltair in the great Salt Lake—inland salt sea nearly a mile above sea-level—is superb in June. There are more attractions in and about Salt Lake City than any place in the world. Later notice will appear in this publication giving rates for this excursion and all details. In the meantime send to F. A. Wadleigh, G. P. A. Rio Grande Western Ry., Salt Lake City, for copy of pamphlets on Salt Lake City and the Rocky Mountains.

---

### INJECTIONS OF ALCOHOL IN CARCINOMA.

Hassel says (*Medical Record,* January 15, 1898):

"Alcohol favors cicatrization in all growths like struma, angioma, cysts, lymphatic-gland tumors, sarcoma, carcinoma, and especially carcinoma of the breast and cervix uteri. Under its use, in fifteen out of eighteen cases of carcinoma of the breast the growth gradually dwindled away, until in a year there was nothing left but the connective-tissue stroma, and there has been no return. Five cases of carcinoma of the cervix also recovered completely, and the patients are still living and in good health. The effect on the general health is even more surprising. The pains and uneasiness pass away, and sleep, appetite, assimilation and strength return in a most remarkable manner.—*Med. Review of Reviews.*

---

### THE HAND IN OBSTETRICS.

The hand as a dilating agent is the best obstetrical instrument at our disposal—better than Barnes's bags and the instruments which have come to us from the French school. As a result of extended experience, I am still able to say that in ninety-eight per cent. of all classes, the woman being within six weeks of full term, and under surgical anesthesia, any man can dilate the cervix with his hand sufficiently to enter the uterus and extract the child.—*Egbert H. Grandin.*

---

### HOW TO MAKE A MUSTARD PLASTER.

Never place a cold mustard plaster on a patient. The shock is like a sudden plunge into cold water. Before you commence to mix the paste be sure you have all the necessary material at hand. First put a large plate where it can get warm, not hot. Then stir the mustard and flour thoroughly together before you add the water, which should be tepid; stir in enough water to make a paste about the consistency of French mustard. Place your cloth (an old handkerchief is best) on the warm plate, spreading the paste in the middle of it, leaving a margin wide enough to lap well over on all sides. Do not remove paste from the plate until ready to apply. Place a folded cloth between paste and patient's clothing.—*Ex.*

---

### DELICATE TEST FOR SUGAR.

| | | |
|---|---|---|
| ℞ | Cupric sulphate | gr. xxvii. |
| | Glycerin | ʒ iii. |
| | Aqua | ʒ iiss. |
| | Liq. potassa | ad. ℥ iv. |

Dissolve the cupric sulphate in glycerine and heat. When cold add the liquor potassa. Pour a drachm of the solution in a test tube with two or three drops of a saturated solution of pure tartaric acid, and boil. Now add, drop by drop, eight drops of urine. If there is no reaction there is no sugar. Sugar is present if the reaction yields a yellowish, reddish, or greenish gray deposit.—*Scientific American.*

---

THE scourge of syphilis has been the most deadly enemy to the upward progress of the human race the world has ever seen. Its disintegrating power in destroying human life has been greater than even pestilence or the sword. Millions upon millions of the human family who have never seen the light of day have been sacrificed *in utero* by embryonic infection to gratify this insatiate moloch of death. Acquired in one generation it insidiously propagates itself to future generations, until who can tell where its baneful influence begins or ends? It masquerades under so many assumed names and in so many unexpected guises that it often baffles and eludes the skill of the most careful diagnostician to locate it. Its peculiar affinity for attacking the brain and nervous system is one of the marked features of its history, and yet it was long before neurologists discovered its potency as a factor in producing insanity. That fatal form of insanity known as general paresis, or as it is now called general paralysis of the insane, is now recognized by high authority as in every case due to this virulent poison. All authorities are agreed that it is the most common cause in producing this and other kindred diseases affecting the brain and nervous system.—*Russell* on the "Relation of Insanity to the State." *Brit. Med. Association, 1897.*

---

### ANTI-NEURALGIA PILLS.

| | | |
|---|---|---|
| ℞ | Strychninæ sulph. .......................................... | gr. i. |
| | Quininæ sulph .......................................... | ʒ i. |
| | Ferri redacti .......................................... | gr. xv. |
| | Ext. Gentianæ.......................................... | ʒ ss. |

M. et ft. pil. No. 60. Sig.—One three times a day.

This is especially good for facial and stomach neuralgias. If there is a marked malarial element present, add arsenious acid, five grains, to the formula.—*Ruff, Med. Rec.*

---

"WE have sent forth a good deal of Seidlitz powder literature—literature which, read by the surgical aspirant, is as fuel to the fires of surgical aggression. While I have the utmost admiration for the bold surgeon, and think the very acme of the world's heroism was attained when Mrs. Crawford clasped hands with McDowell and made her leap for life, my admiration for the bold surgeon warms into fervent flame of love when I learn that his boldness is always tempered by wisdom, and his conscience holds him firmly to the golden rule, 'Whatsoever ye would that men should do unto you, do ye even so unto them.'"—DR. JOSEPH EASTMAN, in *Am. Gyn. and Obs. Jour.*, Feb.

### COUGH MIXTURE.

A cough mixture much employed in the Roosevelt Hospital, New York City, is the following:

| | | |
|---|---|---|
| ℞ | Codeine | 4 gr. |
| | Dil. hydrocyanic acid | 45 drops. |
| | Ammonium chloride | 45 gr. |
| | Syrup wild cherry to make | 1½ fl. oz. |

Teaspoonful every three or four hours.

—*Medico Surgical Bulletin.*

---

IT matters not whether it be the collar, the waistband or the garter, all constricting bands are injurious; anything that compresses the blood-vessels and interferes with the free circulation of the blood should not be tolerated. Headache, chronic digestion of the brain, even apoplexy will be favored and rendered more possible by the use of tight collars; while varicose veins are commonly caused by tight garters. Anything constricting the waist is evidently and obviously injurious to the vital organs within, all of which require plenty of room that they may functionate properly. We think it barbarous for Chinese women to constrict their feet; Li Hung Chang thinks it more barbarous for Americans to constrict their necks, waists and legs. The old man is right; there are no vital organs in the feet.—*Annals of Hygiene.*

---

### TO MAKE A NORMAL SALT SOLUTION FOR TRANSFUSION.

| | | |
|---|---|---|
| ℞ | Sodii carb | gr. xv. |
| | Sodii chlor | ʒ jss. |
| | Aquæ | Oij. |

—*Med. Review.*

---

SARSAPARILLA SAM.—"What you got dat razor fur?"

Pokeberry Pete.—"Yer know dat eddicated nigger wat's teaching school at de big forks? Well, yer see, I owed him a little bill, and he done writ me, he did, dat he gwine ter draw on me at sight."

Sarsaparilla Sam.—"Well, what ob dat?"

Pokeberry Pete.—"Nuttin, 'cept dere ain' no nigger gwine ter git de drap on Pete."—*Atlanta Constitution.*

